# Combined treatment with lexatumumab and irradiation leads to strongly increased long term tumour control under normoxic and hypoxic conditions

**DOI:** 10.1186/1748-717X-4-49

**Published:** 2009-10-27

**Authors:** Patrizia Marini, Dorothea Junginger, Stefan Stickl, Wilfried Budach, Maximilian Niyazi, Claus Belka

**Affiliations:** 1 CCC Tübingen, Dept of Radiation Oncology, University of Tübingen, Hoppe-Seyler-Str 3, 72076 Tübingen, Germany; 2 Dept of Radiation Oncology, LMU University of München, Marchioninistr 15 81377 München, Germany; 3 Dept of Radiation Oncology and Radiotherapy, University of Düsseldorf, Moorenstr 5, 40225 Düsseldorf, Germany

## Abstract

**Purpose:**

The combination of ionizing radiation with the pro-apoptotic TRAIL receptor antibody lexatumumab has been shown to exert considerable synergistic apoptotic effects in vitro and in short term growth delay assays. To clarify the relevance of these effects on local tumour control long-term experiments using a colorectal xenograft model were conducted.

**Materials and methods:**

Colo205-xenograft bearing NMRI (nu/nu) nude mice were treated with fractionated irradiation (5× 3 Gy, d1-5) and lexatumumab (0.75 mg/kg, d1, 4 and 8). The tumour bearing hind limbs were irradiated with graded single top up doses at d8 under normoxic (ambient) and acute hypoxic (clamped) conditions. Experimental animals were observed for 270 days. Growth delay and local tumour control were end points of the study. Statistical analysis of the experiments included evaluation of tumour regrowth and local tumour control.

**Results:**

Combined treatment with irradiation and lexatumumab led to a pronounced tumour regrowth-delay when compared to irradiation alone. The here presented long-term experiments revealed a highly significant rise of local tumour control for normoxic (ambient) (p = 0. 000006) and hypoxic treatment (p = 0. 000030).

**Conclusion:**

Our data show that a combination of the pro-apoptotic antibody lexatumumab with irradiation reduces tumour regrowth and leads to a highly increased local tumour control in a nude mouse model. This substantial effect was observed under ambient and more pronounced under hypoxic conditions.

## Background

Lexatumumab is a fully human agonistic antibody with a distinct tumour cell specifity via activation of TRAIL (TNF-related apoptosis inducing ligand) receptor 2 (TRAIL-R2) induced apoptosis. Although TRAIL-R2 stimulation alone is highly effective in a wide range of cancer cell lines, efficacy can be increased by combination with other gyrostatic drugs (for review see [1]). We have already shown that a combined treatment with TRAIL and irradiation exerts highly synergistic effects regarding apoptosis induction. This enhanced efficacy was detectable in various solid tumour cell lines and lymphoid tumour cells[2,3].

Since discovery of TRAIL and its receptors in 1997 a panel of agonistic antibodies for TRAIL-receptors R1 and R2 have been developed and tested in clinical phase I and II trials [4-18]. However, up to now only little data are available concerning interaction of agonistic TRAIL receptor antibodies and irradiation ([7,19,20]. Besides our recently published report no data on experiments with a combination of a fully human TRAIL receptor antibody and irradiation have been published[21].

Combining mapatumumab or lexatumumab with irradiation, we have demonstrated that this combination exerts strong additive and synergistic effects on apoptosis induction in vitro and in short-term growth delay experiments[10]. However, to proof that induction of apoptosis evidently translates into definitive tumour stem cell eradication long-term experiments with local tumour control as primary endpoint might provide a reliable model for clinical potency [22-26].

Therefore, we decided to perform long-term experiments in a nude mouse xenograft model. As radiation sensitivity becomes affected by limiting intratumoural hypoxia we run experiments under both ambient and hypoxic conditions to mimic realistic tumour conditions[27].

Taken together, our experimental series was designed to confirm the striking principle that radiation mediated TRAIL sensitization effectively increases long-term local tumour control.

## Materials and methods

### Animals and tumours

Immunodeficient NMRI-(nu/nu)-nude mice were purchased from a specific pathogen free colony at the University of Essen (Germany) at the age of 4-6 weeks. Animals were kept in an individually ventilated cage rack system (Techniplast, Italy) and fed with sterile high calorie laboratory food (Sniff, Germany). Drank water was supplemented by chlorotetracycline and potassium sorbate acidified to a pH of 3.0 with hydrochloric acid.

The Colo205 tumour cell line (established from a colorectal adenocarcinoma) was acquired from ATCC (Bethesda, MD, USA). In NMRI-(nu/nu)-nude mice Colo205 cells form solid, roundly shaped tumours without indication for metastasis.

### Transplantation and experimental design

Tumour lumps of about 2 mm diameter from a source tumour were implanted subcutaneously into the right hind limb of 6-10 week old animals. Approximately 2-3 weeks after transplantation tumour growth was measurable. Tumour size was quantified with calipers in two perpendicular diameters. The tumour volume (V) was calculated as V = (a × b^2^)/2, where a and b are the long axis and the short axis, respectively. Scoring of tumour sizes took place three times per week before start of treatment. Body weight was monitored once a week.

**Figure 1:**
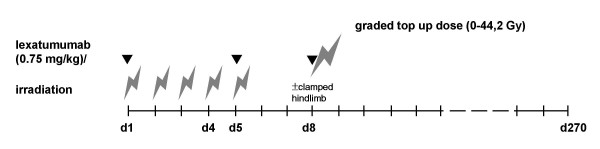
**Experimental design**. Small bolt = fractionated irradiation at d 1-5, large bolt = graded top up doses 0-44.2 Gy (under ambient/hypoxic conditions, depending on stratification), small arrowhead: application of lexatumumab (0.75 mg/kg body weight), d = day.

The median tumour volume at the start of experiments was 116 ± 31 mm^3^. Animals were randomly allocated to 24 treatment arms (scheme see Figure 1): lexatumumab at day 1, 4 and 8 (0.75 mg/kg body weight intraperitoneally (i.p.)) alone, fractioned radiotherapy (5 × 3 Gy within five subsequent days) alone. Single dose top up irradiations (0, 10.0, 14.5, 21.0, 30.4, 44.2 Gy) were performed on day 8. Combined treatment was performed at day 1, 4 and 8 with lexatumumab (0.75 mg/kg) (figure 1). Control animals were treated only with an i.p. injection of medium without antibody or irradiation.

To minimize toxic side effects and to apply high irradiation doses in an easy comparable, time saving schedule we choose a combination of fractionated and graded single high dose (top up) irradiation. 3 Gy single dose was chosen for fractionated irradiation based on previous experiments (Marini et al., Oncogene 2006). Fractionated irradiation of tumours was applied in inhalation (Isoflurane) narcosis. Top up irradiation under ambient conditions or under clamped hypoxia was performed with i.p. narcosis (fentanyl, midazolam, medetomidine), as recommended by the university veterinarian department. For animals, whose tumours were clamped irradiation was performed 10 minutes after applying a narrow lace to the right hind limb just at the proximal end of the tumour to make the hypoxic radiation conditions as consistent as possible. Experiments were performed in one run with 252 animals.

Tumour volumes were scored twice a week, no blinding took place. Follow up was discontinued after 270 days or in case of intercurrent death or if tumours had grown to eight-times the initial tumour volume at the start of treatment. Growth delay and local tumour control were endpoints of the study. All animal experiments were accomplished in accordance with the guidelines of the local authorities (Regional Board Tuebingen, Germany, appl.no. R4/04) and the German animal welfare regulations.

### Statistical Analysis

Statistical analysis was performed as described before[21]. In short terms, an exponential regression model was used to interpolate median tumour regrowth times. Regrowth delay was compared by unparametric Kruskal-Wallis tests with Dunn's post tests. Tumour control rates were calculated accounting for censored animals as described by Walker and Suit[28]. Data were analysed by a probit non linear regression analysis. Parameters were estimated using the maximum likelihood method. Statistical significance was calculated asymptotically by means of a Hessian matrix (STATISTICA 6.0 StatSoft, Hamburg, Germany).

## Results

Treatment with lexatumumab failed to induce any immune reactions of the irradiated skin. No evidence of acute toxicity was observed. Follow up revealed no significant differences in frequency of intercurrent deaths after irradiation alone or combined treatment with lexatumumab (5.6% vs. 4.6%).

**Figure 2:**
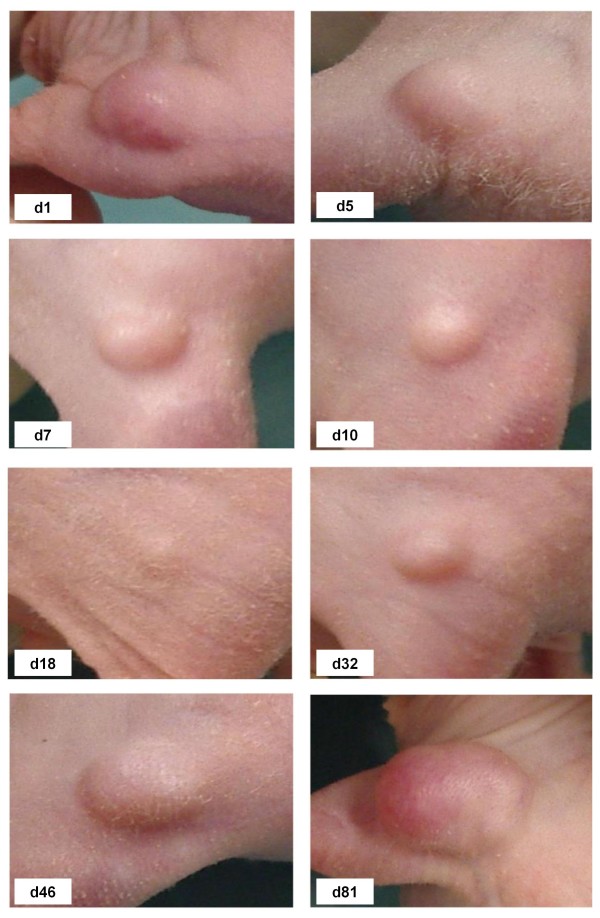
**Photographic showcase of the chronological sequence of tumour regression and tumour regrowth after i.p. application of lexatumumab (0.75 mg/kg; d 1, 4 and 8) from day 1(d1) up to day 81 (d81) of treatment**.

Figure 2 shows a chronological sequence of the impressive tumour regression after treatment with lexatumumab (0.75 mg/kg) for one test animal, exemplarily. Obviously, tumour growth reduction started after the second application i.p., already. However, lacking consolidating irradiation in this example tumour regrowth is evident four weeks after start of treatment.

**Figure 3:**
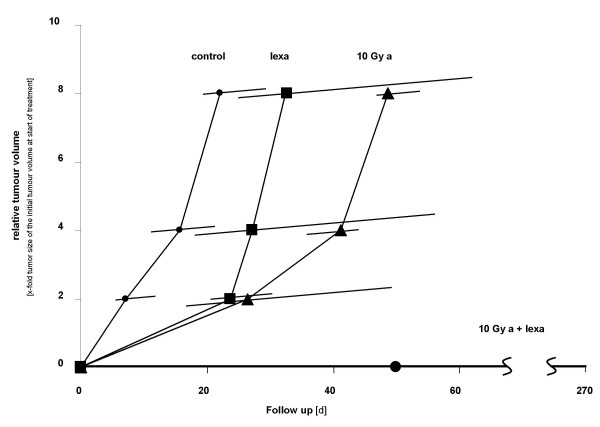
**Median tumour regrowth times, calculated for two-, four-, and eight-fold tumour size of the initial tumour volume at start of treatment**. Crossbars show 25-75% quartiles for each tumour volume and each treatment. Control; small circle, solid line = animals receiving only i.p. injection with medium, without any further treatment. 10 Gy, square, solid line = fractionated irradiation (3 × 5 Gy) + 10 Gy single top up irradiation. Lexa; triangle, solid line = lexatumumab (0.75 mg/kg body weight, i.p. injection d 1, 4, 8). 10 Gy + lexa; large circle, solid line = fractionated irradiation (3 × 5 Gy) + 10 Gy single top up irradiation and lexatumumab (0.75 mg/kg body weight, i.p. injection d 1, 4, 8). a = Treatment under ambient conditions.

However, combination of very low doses of irradiation with lexatumumab led to an unexpected high local tumour rate, already. Tumour regrowth after combined treatment was observed in less than 50% of the animals. Figure 3 shows data on the 2-, 4- and 8-fold tumour regrowth after single and combined treatment with a 10 Gy top up dose, exemplarily. In this subset of experiments, five of nine mice were lacking any tumour regrowth 270 days after start of treatment. Analysis of the median time of tumour regrowth after combined treatment was impaired by an unexpected high rate of local control (figure 3). Therefore, we decided to choose the more complex probit non linear regression analysis.

**Figure 4:**
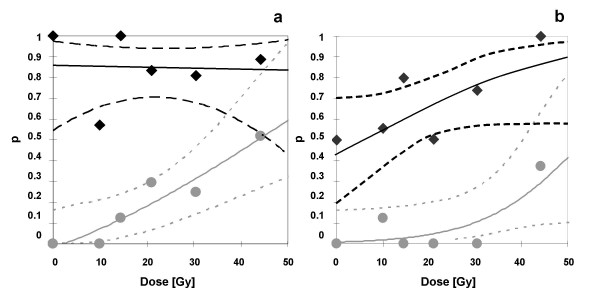
**Dose-response relation between tumour control probability and top up irradiation dose for Colo205 xenograft tumours**. Grey circle, solid grey line = tumours treated with fractionated irradiation (5 × 3 Gy) and graded single top up doses (0-44.2 Gy) alone. Black diamond, solid black line = tumours treated with fractionated irradiation (5 × 3 Gy) and graded single top up doses (0-44.2 Gy) and lexatumumab (0.75 mg/kg body weight, i.p. injection d 1, 4, 8) a: under ambient conditions, b: under hypoxic conditions. Dashed lines represent the 95% confidence level.

Figure 4 depicts the extraordinary efficacy of the combined treatment by the probit analysis. Irradiation with graded top up doses from 0 to 44.2 Gy alone resulted in local tumour control from 0 to 52% under ambient conditions (figure 4a, grey solid line). Addition of lexatumumab after fractionated irradiation alone already caused very high tumour control rates of 85-87%, regardless of the top up dose (p = 0.000006, figure 4a, black solid line). Under clamped bloodflow, treatment with lexatumumab enhanced local tumour control after irradiation with fractionated irradiation and graded top up doses (0 to 44.2 Gy) alone from 0% - 30% (figure 4b, grey solid line) up to 43 - 87% (p = 0.00003, figure 4b, black solid line). Statistical analysis unveiled a highly significant increase of tumour control rates under both, ambient (p < 0.0001) and hypoxic (p < 0.0001) conditions (table 1).

**Table 1:** Results of the probit regression analysis comparing combined treatment (lexatumumab (= lexa, 0.75 mg/kg) and irradiation (= RT, 5 × 3 Gy and graded top up doses 0-44.2 Gy) with irradiation alone

	**const. B0^#^**	**RT-dose (B1)**	**lexa (B2)**
***normoxia***			

Parameter (MLE*)	- 1.729	0.028	2.062
**SE^§^**	0.386	0.012	0.343
**p-value**	0.0002	0.0294	<0.0001

***clamped hypoxia***			

**Parameter (MLE)**	- 2.424	0.035	2.097
**SE**	0.489	0.013	0.396
**p-value**	<0.0001	0.0147	<0.0001

^# ^Regression constant B0

* Maximum likelihood estimate

**§ **Standard error

## Discussion

Our data prove that the combination of the proapoptotic human antibody lexatumumab with ionizing radiation has an obvious influence on local tumour control in a long-term xenograft model. The effect is evident after irradiation with low doses, already.

It is important to note that these experiments with an agonistic antibody against TRAIL receptor DR5 corroborate our recently published data on a high efficacy of a combined treatment with another proapoptotic antibody (mapatumumab, anti-DR4) and irradiation. Both models are in line with in vitro data from our and other labs demonstrating that irradiation acts as a TRAIL sensitizer and not obversely[3,29,30].

This principle diverges from other combined approaches where classical chemotherapeutic or other molecular targeted agents act as radiosensitizer. E.g. the synergizing efficacy of cisplatin is based on increased oxygenation of hypoxic cells and an influence in DNA-repair and cell cycle regulation [31-33]. Cetuximab, an antibody against epidermal growth factor receptor, seems also to influence long-term tumour control by affecting DNA damage repair[34,35].

In contrast to former reports the mitochondrial pathway has a strong impact in TRAIL induced apoptosis. Depending on the cell system applied mitochondrial amplification loops account for its high efficacy[36,37]. In combination with TRAIL, irradiation increases apoptosis in tumour cells with an impaired mitochondrial pathway. Furthermore, preirradiation of bcl-2 overexpressing lymphoma cells raises cell death rates after TRAIL receptor stimulation[38]. In several tumour cell systems, the proapoptotic molecule Bax was shown to be essential for the combined effect of TRAIL and ionizing radiation suggesting a considerable mitochondrial relevance for this synergizing principle[10,39,40].

The role of radiation induced TRAIL receptor upregulation has been discussed extensively. However, we and others found an only weak or lacking correlation between upregulation and synergism [10,41,42]. Although, other mechanisms like cell cycle regulation might play a role [43].

It is important to note, that this synergistic principle works under ambient and hypoxic conditions as well. Weinmann et al. demonstrated an undiminished efficacy of TRAIL alone under hypoxia in a lymphoma cell model[44]. Takahashi at al. reported similar observations on clonogenic cell kill of A549 cells after treatment with TRAIL and irradiation[45]. However, it remains speculative why this effect on local tumour control is more pronounced under normoxia than under hypoxia. The known increase of intrinsic radioresistance of hypoxic cells will be responsible for this reduced susceptibility.

The strong request on the development of personalized targeted therapies has amazingly changed the general approach to cancer treatment. In contrast to cytostatic drugs being prescribed on base of classical features as TNM classification and histology, targeted drugs require an accurate identification of patient collectives who benefit from a given treatment. Therefore, a specific subset of marker molecules should be identified for each targeted drug [46-48].

## Conclusion

The here presented data provide evidence that the combination of apoptosis inducing antibodies with irradiation strongly increases long-term tumour control. Since murine long-term control experiments are the only currently accepted functional approach to simulate the efficacy of radiation based treatments the given data are an optimal scientific base for subsequent clinical trials.

## Competing interests

The authors declare that they have no competing interests.

## Authors' contributions

PM conceived and drafted the manuscript. DJ and SS carried out the animal experiments to the same portion. WB performed the statistical analysis. MN participated in the statistical analysis and in the drafting of the manuscript. CB contributed to interpretation of the data and critically reviewed the article. All authors read and approved the final manuscript.

## References

[B1] Ashkenazi A, Holland P, Eckhardt SG (2008). Ligand-based targeting of apoptosis in cancer: The potential of recombinant human apoptosis ligand 2/tumor necrosis factor-related apoptosis-inducing ligand (rhapo2l/trail). J Clin Oncol.

[B2] Belka C, Schmid B, Marini P, Durand E, Rudner J, Faltin H, Bamberg M, Schulze-Osthoff K, Budach W (2001). Sensitization of resistant lymphoma cells to irradiation-induced apoptosis by the death ligand trail. Oncogene.

[B3] Marini P, Schmid A, Jendrossek V, Faltin H, Daniel PT, Budach W, Belka C (2005). Irradiation specifically sensitises solid tumour cell lines to trail mediated apoptosis. BMC Cancer.

[B4] Pan G, O'Rourke K, Chinnaiyan AM, Gentz R, Ebner R, Ni J, Dixit VM (1997). The receptor for the cytotoxic ligand trail. Science.

[B5] Walczak H, Miller RE, Ariail K, Gliniak B, Griffith TS, Kubin M, Chin W, Jones J, Woodward A, Le T, Smith C, Smolak P, Goodwin RG, Rauch CT, Schuh JC, Lynch DH (1999). Tumoricidal activity of tumor necrosis factor-related apoptosis-inducing ligand in vivo. Nat Med.

[B6] Camidge DR (2008). An agonist monoclonal antibody directed against death receptor 5/trail-receptor 2 for use in the treatment of solid tumors. Expert Opin Biol Ther.

[B7] Fiveash JB, Gillespie GY, Oliver PG, Zhou T, Belenky ML, Buchsbaum DJ (2008). Enhancement of glioma radiotherapy and chemotherapy response with targeted antibody therapy against death receptor 5. Int J Radiat Oncol Biol Phys.

[B8] Humphreys R (2004). HGS-TR2J, a human, agonistic, trail receptor-2 monoclonal antibody, induces apoptosis, tumor regression and growth inhibition as a single agent in diverse human solid tumor cell lines. Abstract #204: 16th EORTC-NCI-AACR Symposium on Molecular Targets and Cancer Therapeutics Genevre, Swiss.

[B9] Ichikawa K, Liu W, Zhao L, Wang Z, Liu D, Ohtsuka T, Zhang H, Mountz JD, Koopman WJ, Kimberly RP, Zhou T (2001). Tumoricidal activity of a novel anti-human dr5 monoclonal antibody without hepatocyte cytotoxicity. Nat Med.

[B10] Marini P, Denzinger S, Schiller D, Kauder S, Welz S, Humphreys R, Daniel PT, Jendrossek V, Budach W, Belka C (2006). Combined treatment of colorectal tumours with agonistic trail receptor antibodies HGS-ETR1 and HGS-ETR2 and radiotherapy: Enhanced effects in vitro and dose-dependent growth delay in vivo. Oncogene.

[B11] Mom CH, Sleijfer S, Gietema JA, Fox NL, Piganeau C, Lo L, Uges DRA, Loos W, de Vries EGE, Verweij J (2006). Mapatumumab, a fully human agonistic monoclonal antibody that targets TRAIL-R1, in combination with gemcitabine and cisplatin: A phase 1b study in patients with advanced solid malignancies.

[B12] Motoki K, Mori E, Matsumoto A, Thomas M, Tomura T, Humphreys R, Albert V, Muto M, Yoshida H, Aoki M, Tamada T, Kuroki R, Yoshida H, Ishida I, Ware CF, Kataoka S (2005). Enhanced apoptosis and tumor regression induced by a direct agonist antibody to tumor necrosis factor-related apoptosis-inducing ligand receptor 2. Clin Cancer Res.

[B13] Pacey S, Plummer RE, Attard G, Bale C, Calvert AH, Blagden S, Fox NL, Corey A, de Bono JS (2005). Phase I and pharmacokinetic study of HGS-ETR2, a human monoclonal antibody to TRAIL R2, in patients with advanced solid malignancies. J Clin Oncol.

[B14] Saleh MN, Percent I, Wood TE, Posej J, Shah J, Carlisle R, Wojtowicz-Praga S, Forero-Torres A (2008). A phase I study of CS-1008 (humanized monoclonal antibody targeting death receptor 5 or DR5), administered weekly to patients with advanced solid tumors or lymphomas.ASCO Annual meeting. Orlando, Florida, USA,. J Clin Oncol.

[B15] Sikic BI, Wakelee H, von Mehren M, Lewis NL, Plummer ER, Calvert AH, Fox NL, Kumm EA, Jones DF, Burris HA A phase 1b study to assess the safety of lexatumumab, a human monoclonal antibody that activates TRAIL-R2, in combination with gemcitabine, pemetrexed, doxorubicin or FOLFIRI. Abstract, 2007.. Proceedings of the American Society of Clinical Oncology.

[B16] Tolcher AW, Mita M, Meropol NJ, von Mehren M, Patnaik A, Padavic K, Hill M, Mays T, McCoy T, Fox NL, Halpern W, Corey A, Cohen RB (2007). Phase I pharmacokinetic and biologic correlative study of mapatumumab, a fully human monoclonal antibody with agonist activity to tumor necrosis factor-related apoptosis-inducing ligand receptor-1. J Clin Oncol.

[B17] Vulfovich M, Saba N (2005). Mapatumumab, human genome sciences/glaxosmithkline/takeda. Curr Opin Mol Ther.

[B18] Younes A, Vose JM, Zelenetz AD, Smith MR, Burris H, Ansell S, Klein J, Kumm E, Czuczman M (2005). Results of a phase 2 trial of HGS-ETR1 (agonistic human monoclonal antibody to TRAIL receptor 1) in subjects with relapsed/refractory non-hodgkin's lymphoma (NHL). Blood.

[B19] Straughn JM, Oliver PG, Zhou T, Wang W, Alvarez RD, Grizzle WE, Buchsbaum DJ (2006). Anti-tumor activity of tra-8 anti-death receptor 5 (DR5) monoclonal antibody in combination with chemotherapy and radiation therapy in a cervical cancer model. Gynecol Oncol.

[B20] Buchsbaum DJ, Zhou T, Grizzle WE, Oliver PG, Hammond CJ, Zhang S, Carpenter M, LoBuglio AF (2003). Antitumor efficacy of tra-8 anti-DR5 monoclonal antibody alone or in combination with chemotherapy and/or radiation therapy in a human breast cancer model. Clin Cancer Res.

[B21] Marini P, Budach W, Niyazi M, Junginger D, Stickl S, Jendrossek V, Belka C (2009). Combination of the pro-apoptotic trail-receptor antibody mapatumumab with ionizing radiation strongly increases long term tumor control under ambient and hypoxic conditions. Int J Radiat Oncol Biol Phys.

[B22] Baumann M, Krause M, Zips D, Eicheler W, Dorfler A, Ahrens J, Petersen C, Bruchner K, Hilberg F (2003). Selective inhibition of the epidermal growth factor receptor tyrosine kinase by BIBX1382BS and the improvement of growth delay, but not local control, after fractionated irradiation in human fadu squamous cell carcinoma in the nude mouse. Int J Radiat Biol.

[B23] Borst P, Borst J, Smets LA (2001). Does resistance to apoptosis affect clinical response to antitumor drugs?. Drug Resist Updat.

[B24] Brown JM, Wouters BG (1999). Apoptosis, p53, and tumor cell sensitivity to anticancer agents. Cancer Res.

[B25] Krause M, Prager J, Zhou X, Yaromina A, Dorfler A, Eicheler W, Baumann M (2007). EGFR-TK inhibition before radiotherapy reduces tumour volume but does not improve local control: Differential response of cancer stem cells and nontumourigenic cells?. Radiother Oncol.

[B26] Schmitt CA, Lowe SW (2001). Apoptosis is critical for drug response in vivo. Drug Resist Updat.

[B27] Harris AL (2002). Hypoxia-a key regulatory factor in tumour growth. Nat Rev Cancer.

[B28] Walker AM, Suit HD (1983). Assessment of local tumor control using censored tumor response data. Int J Radiat Oncol Biol Phys.

[B29] Shankar S, Singh TR, Chen X, Thakkar H, Firnin J, Srivastava RK (2004). The sequential treatment with ionizing radiation followed by trail/apo-2l reduces tumor growth and induces apoptosis of breast tumor xenografts in nude mice. Int J Oncol.

[B30] Shankar S, Singh TR, Srivastava RK (2004). Ionizing radiation enhances the therapeutic potential of trail in prostate cancer in vitro and in vivo: Intracellular mechanisms. Prostate.

[B31] Douple EB, Richmond RC (1979). Radiosensitization of hypoxic tumor cells by cis- and trans-dichlorodiammineplatinum (II). Int J Radiat Oncol Biol Phys.

[B32] Hoebers FJ, Pluim D, Verheij M, Balm AJ, Bartelink H, Schellens JH, Begg AC (2006). Prediction of treatment outcome by cisplatin-DNA adduct formation in patients with stage III/IV head and neck squamous cell carcinoma, treated by concurrent cisplatin-radiation (radplat). Int J Cancer.

[B33] Chu G (1994). Cellular responses to cisplatin. The roles of DNA-binding proteins and DNA repair. J Biol Chem.

[B34] Dittmann K, Mayer C, Rodemann HP (2005). Inhibition of radiation-induced egfr nuclear import by c225 (cetuximab) suppresses DNA-PK activity. Radiother Oncol.

[B35] Huang SM, Harari PM (2000). Modulation of radiation response after epidermal growth factor receptor blockade in squamous cell carcinomas: Inhibition of damage repair, cell cycle kinetics, and tumor angiogenesis. Clin Cancer Res.

[B36] Suliman A, Lam A, Datta R, Srivastava RK (2001). Intracellular mechanisms of trail: Apoptosis through mitochondrial-dependent and -independent pathways. Oncogene.

[B37] Cuello M, Coats AO, Darko I, Ettenberg SA, Gardner GJ, Nau MM, Liu JR, Birrer MJ, Lipkowitz S (2004). N-(4-hydroxyphenyl) retinamide (4HPR) enhances trail-mediated apoptosis through enhancement of a mitochondrial-dependent amplification loop in ovarian cancer cell lines. Cell Death Differ.

[B38] Belka C, Schmid B, Marini P, Durand E, Rudner J, Faltin H, Bamberg M, Schulze-Osthoff K, Budach W (2001). Sensitization of resistant lymphoma cells to irradiation-induced apoptosis by the death ligand TRAIL. Oncogene.

[B39] von Haefen C, Gillissen B, Hemmati PG, Wendt J, Guner D, Mrozek A, Belka C, Dorken B, Daniel PT (2004). Multidomain Bcl-2 homolog bax but not Bak mediates synergistic induction of apoptosis by TRAIL and 5-FU through the mitochondrial apoptosis pathway. Oncogene.

[B40] Deng Y, Lin Y, Wu X (2002). TRAIL-induced apoptosis requires Bax-dependent mitochondrial release of smac/diablo. Genes Dev.

[B41] Griffith TS, Rauch CT, Smolak PJ, Waugh JY, Boiani N, Lynch DH, Smith CA, Goodwin RG, Kubin MZ (1999). Functional analysis of TRAIL receptors using monoclonal antibodies. J Immunol.

[B42] Luciano F, Ricci JE, Herrant M, Bertolotto C, Mari B, Cousin JL, Auberger P (2002). T and B leukemic cell lines exhibit different requirements for cell death: Correlation between caspase activation, dff40/dff45 expression, DNA fragmentation and apoptosis in T cell lines but not in Burkitt's lymphoma. Leukemia.

[B43] Wu F, Hu Y, Long J, Zhou YJ, Zhong YH, Liao ZK, Liu SQ, Zhou FX, Zhou YF, Xie CH (2009). Cytotoxicity and radiosensitization effect of TRA-8 on radioresistant human larynx squamous carcinoma cells. Oncol Rep.

[B44] Weinmann M, Marini P, Jendrossek V, Betsch A, Goecke B, Budach W, Belka C (2004). Influence of hypoxia on TRAIL-induced apoptosis in tumor cells. Int J Radiat Oncol Biol Phys.

[B45] Takahashi M, Inanami O, Kubota N, Tsujitani M, Yasui H, Ogura A, Kuwabara M (2007). Enhancement of cell death by TNF alpha-related apoptosis-inducing ligand (TRAIL) in human lung carcinoma a549 cells exposed to x rays under hypoxia. J Radiat Res (Tokyo).

[B46] Sturm I, Rau B, Schlag PM, Wust P, Hildebrandt B, Riess H, Hauptmann S, Dorken B, Daniel PT (2006). Genetic dissection of apoptosis and cell cycle control in response of colorectal cancer treated with preoperative radiochemotherapy. BMC Cancer.

[B47] Mrozek A, Petrowsky H, Sturm I, Kraus J, Hermann S, Hauptmann S, Lorenz M, Dorken B, Daniel PT (2003). Combined p53/Bax mutation results in extremely poor prognosis in gastric carcinoma with low microsatellite instability. Cell Death Differ.

[B48] Kallioniemi A (2008). CGH microarrays and cancer. CurrOpin Biotechnol.

